# Diagnostic Test Accuracy of Genetic Tests in Diagnosing Psoriasis: A Systematic Review

**DOI:** 10.7759/cureus.31338

**Published:** 2022-11-10

**Authors:** Hyder Mirghani, Abdulrahman Arshed N Alharfy, Abeer Mohammed M Alanazi, Jomanah Khalid M Aljohani, Raghad Abdulrahman A Aljohani, Raghad Hamdan A Albalawi, Raneem Abdulrahman A Aljohani, Danah Mohsen Alqasmi Albalawi, Rahaf Hamdan A Albalawi, Mohamed I Mostafa

**Affiliations:** 1 Department of Internal Medicine, Faculty of Medicine, University of Tabuk, Tabuk, SAU; 2 Department of Dermatology, Faculty of Medicine, University of Tabuk, Tabuk, SAU; 3 Department of Anatomy, Faculty of Medicine, University of Tabuk, Tabuk, SAU

**Keywords:** risk score, psoriasis, prediction, genetic testing, diagnostic accuracy

## Abstract

The pathogenesis of psoriasis involves the interaction of several environmental and genetic factors. Predicting the disease risk cannot depend on individual genetic alleles. Consequently, some studies have evaluated the use of genetic risk scores that combine several psoriasis susceptibility loci to increase the accuracy of predicting/diagnosing the disease. This meta-analysis summarizes the evidence regarding using genetic risk scores (GRS) in the diagnosis or prediction of psoriasis. A search of MEDLINE/PubMed, the Latin American Caribbean Health Sciences Literature (LILACS) database, Cochrane Library, Scopus, Web of Science, and ProQuest was conducted in July 2022. The primary objective was to record the area under the curve (AUC) for GRS of psoriasis. Secondary objectives included characteristics of studies and patients. The risk of bias (ROB) was assessed using the PROBAST tool. Five studies fulfilled the eligibility criteria of this review. None of the studies described the clinical criteria (reference standard) that were employed to diagnose psoriasis. The AUCs of the 11 GRS models ranged from 0.6029-0.8583 (median: 0.75). Marked heterogeneity was detected (Cochran Q: 1250.051, p < 0.001, and I^2^ index: 99.2%). So, pooling of the results of the included studies was not performed. The ROB was high for all studies and clinical application was not described. Genetic risk scores are promising tools for the prediction of psoriasis with fair to good accuracy. However, further research is required to identify the most accurate combination of loci and to validate the scores in variable ethnicities.

## Introduction and background

Psoriasis is a chronic inflammatory autoimmune skin disease that affects 0.2% to 6.5% of the general population worldwide [1]. Psoriasis can present in various forms. The two main clinical categories include non-pustular and pustular psoriasis. Non-pustular psoriasis comprises subphenotypes such as psoriasis vulgaris, guttate, erythrodermic, palmoplantar, psoriatic arthritis, and inverse psoriasis. Pustular psoriasis manifests as generalized pustular psoriasis, impetigo herpetiformis or localized pustular psoriasis. The most prevalent form is psoriasis vulgaris which produces well-defined erythematous plaques covered with silvery scales [2].

The diagnosis of psoriasis in clinical practice entails recognition of the distribution, configuration, and morphology of skin lesions. The reference standard for psoriasis diagnosis is a clinical examination by a qualified dermatologist. Clinical diagnostic criteria are not routinely used for diagnosing psoriasis [3].

Psoriasis vulgaris is a genetically complex disease for which research has identified approximately 65 susceptibility loci [4]. The genes located in these loci contribute to the pathogenesis of the disease by mediating the pathways of epidermal differentiation (LCE3B, LCE3C), interleukins 12 and 23 (IL12, IL23A, IL23R, TRAF3IP2, TYK2), nuclear factor-kappa B and interferons signalling (TNFAIP3, TNIP1, NFKBIA, REL, TYK2, IFIH1, IL23RA), T helper 2 cells (IL4, IL13) and CD8 T cells (ERAP1, ZAP70) [5].

The use of individual susceptibility loci for computing psoriasis risk prediction yields insufficiently accurate results. The combination of multiple risk alleles into a genetic risk score (GRS) may improve the prediction of psoriasis and the diagnostic accuracy when definite criteria are required for including patients in clinical trials or observational studies [3,6]. This systematic review aimed to summarize the evidence regarding using GRS in the diagnosis or prediction of psoriasis.

## Review

Materials and methods

Methodology

This systematic review was conducted according to the principles of the Cochrane Handbook for Systematic Reviews of Diagnostic Test Accuracy. Reporting of the results followed the Preferred Reporting Items for Systematic Reviews and Meta-Analyses (PRISMA) guideline [7].

The research question was to determine the diagnostic accuracy of GRS for diagnosing psoriasis in patients with cutaneous psoriasis. This systematic review aimed at summarizing the evidence regarding the diagnostic accuracy of GRS in diagnosing/predicting psoriasis, with the following objectives: (a) to assess the diagnostic accuracy of different genetic tests and scores for diagnosing psoriasis and (b) to investigate potential sources of heterogeneity as differences across age groups or ethnicities.

Cross-sectional and case-control studies were included in this systematic review. The search was restricted to studies published in English during the period from inception to the 1st of July 2022. The included studies were conducted on patients with psoriasis besides a control group of healthy subjects. The index tests were genetic risk scores for psoriasis that combine more than one susceptibility loci, and the target conditions were patients with psoriasis irrespective of the psoriasis subtypes. The reference standards were the history combined with clinical examination with or without skin biopsy. Excluded types of publications comprised longitudinal studies, conference abstracts/posters, duplicate reports, case reports, narrative review articles, editorials, commentaries, and clinical guidelines. In addition, studies published only in languages other than English were excluded.

Search Methods for Identification of Studies

A search of the electronic databases of MEDLINE/PubMed, the Latin American Caribbean Health Sciences Literature (LILACS) database, Cochrane Library, Scopus, Web of Science, and ProQuest was conducted. The search was limited to studies published in English from inception to the 1st of July 2022. The search was conducted during the period from the 26th of June 2022 to the 1st of July 2022. The used search terms were "Psoriasis diagnosis" AND "Genetic Testing". In addition, a search was conducted for potentially relevant studies that were identified from the reference lists of studies retrieved from the electronic search.

Data Collection

Three reviewers carried out the research and then screened the titles and abstracts of retrieved studies. The full text of the studies with potentially relevant abstracts was obtained and screened for eligibility by the same reviewer using the aforementioned inclusion and exclusion criteria. The fourth, fifth, and sixth reviewers checked the results of the search, the screening process of titles and abstracts as well as the review of full text of potentially eligible studies. Any disagreement was settled by the seventh, eighth, and ninth reviewers.

Three reviewers extracted relevant data from the included studies using a standardized datasheet. The extracted data included: (a) the study characteristics (study design, the ethnicity of participants, the number of patients and control subjects); (b) patients’ characteristics (age, sex, and family history); (c) the reference standard for diagnosing psoriasis; (d) the GRS (used loci and method of calculation); (e) diagnostic accuracy measures (area under the receiver operating characteristic curve, sensitivity, or specificity); and (f) association of the GRS with age at onset. The fourth, fifth, and sixth reviewers checked the consistency and clarity of the collected data. Disagreements were resolved by consulting the seventh, eighth, and ninth reviewers. No blinding was used for the journal titles, authors, or institutions.

Assessment of Methodological Quality

Quality assessment of the risk of bias (ROB) and applicability was conducted using the Prediction model Risk of Bias Assessment Tool (PROBAST) [8]. The tool is composed of 20 signaling questions categorized into four main domains (participants, predictors, outcome, and analysis). The signaling questions are rated as “yes”, “probably yes”, “probably no”, “no” or “no information”. Answering the signaling question with “yes” or “probably yes” indicates a lack of ROB. On the other hand, answering with “no” or “probably no” indicates a potential ROB. Each domain is then rated to have low, high or unclear ROB. Concerns for applicability are rated for the first three domains (participants, predictors, and outcome), to indicate whether high, low, or unclear concerns exist. The assessment was performed by the three reviewers and the fourth, fifth, and sixth reviewers revised the results. Disagreements were resolved by consulting the seventh, eighth, and ninth reviewers.

Data Synthesis

MedCalc Statistical Software version 15.8 (MedCalc Software Ltd, Ostend, Belgium; https://www.medcalc.org; 2015) was used for creating the forest plot and testing heterogeneity. A forest plot was created for the reported area under the receiver operating characteristics curves (AUCs) of GRS. The publication bias of studies was assessed using a funnel plot. Heterogeneity testing was performed using the Cochran chi-square heterogeneity test and I^2^ index. Significant heterogeneity across the studies was determined at a Cochran chi-square test with a p-value < 0.1 and an I^2^ index ≥ 50%.

Results

Results of Search

**Figure 1:**
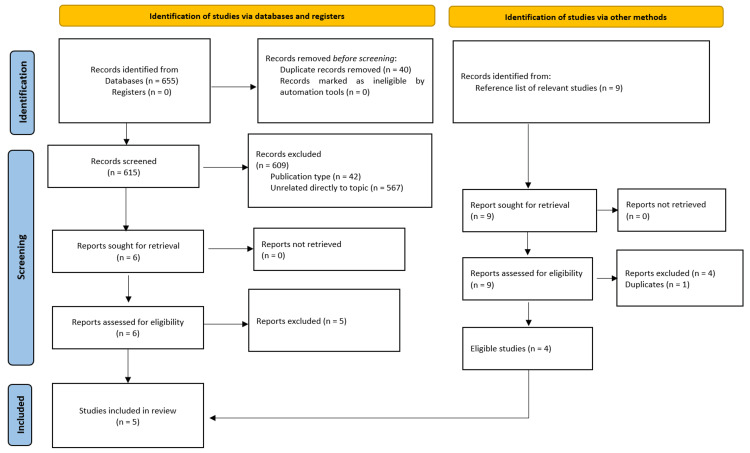
The PRISMA flow chart diagram for the results of the literature search and study selection. PRISMA: Preferred Reporting Items for Systematic Reviews and Meta-Analyses; n: number

The PRISMA flowchart illustrates the results of the literature search, as can be seen in Figure 1. Forty duplicate results were removed before screening the title and abstract of the remaining 615 articles. Forty-two results belonged to the non-eligible types of publications for the current systematic review (35 reviews, three conference abstracts, one case report, one report, one correction, and one editorial article). In addition, 567 articles were non-relevant to the review question (did not include a calculation of a GRS or scores were calculated for other skin diseases). The full text of the remaining six articles was retrieved. Scrutinization of the reference lists of relevant reviews and other studies retrieved another nine articles, out of which one was a duplicate. After applying the eligibility criteria of this systematic review to the full texts of the 14 studies, only five studies were included in the review [6,9-12]. The reasons for the exclusion of the eight studies are listed in Table 1 [4,13-20].

**Table 1:** The excluded studies after full-text retrieval. GRS: genetic risk score; SNP: single nucleotide polymorphism; HLA: human leucocyte antigen

First author (year)	Title	Journal	DOI	Causes for exclusion
Kamsteeg (2010) [16]	Molecular diagnostics of psoriasis, atopic dermatitis, allergic contact dermatitis and irritant contact dermatitis	Br J Dermatol	10.1111/j.1365-2133.2009.09547.x.	ethnicity and calculation method of GRS was unclear
Fang (2011) [13]	Psoriasis prediction from genome-wide SNP profiles	BMC Dermatology	10.1186/1471-5945-11-1	non-relevant (no GRS)
Guo (2014) [14]	Gene expression profile-based classification models of psoriasis	Genomics	10.1016/j.ygeno.2013.11.001	ethnicity was not stated, and the calculation method of GRS was unclear
Inkeles (2015) [15]	Comparison of molecular signatures from multiple skin diseases identifies mechanisms of immunopathogenesis	J Invest Dermatol	10.1038/jid.2014.352	ethnicity was not stated, and the calculation method of GRS was unclear
Sundarrajan (2016) [18]	Weighted gene co-expression-based biomarker discovery for psoriasis detection	Gene	10.1016/j.gene.2016.08.021	ethnicity was not stated, and the calculation method of GRS was unclear
Zhang (2017) [20]	Investigation of 36 non-HLA (human leucocyte antigen) psoriasis susceptibility loci in a psoriatic arthritis cohort	Archives of Dermatological Research	10.1007/s00403-016-1706-z	non-relevant (no GRS)
Tsoi (2017) [4]	Large scale meta-analysis characterizes genetic architecture for common psoriasis-associated variants	Nature Communications	10.1038/ncomms15382	details of constructing GRS are not available
Smith (2020) [17]	Evaluation of a Genetic Risk Score for Diagnosis of Psoriatic Arthritis	Journal of Psoriasis and Psoriatic Arthritis	10.1177/2475530320910814	the GRS is for psoriatic arthritis
Tapak (2021) [19]	Application of Genetic Algorithm-Based Support Vector Machine in Identification of Gene Expression Signatures for Psoriasis Classification: A Hybrid Model	Biomed Research International	10.1155/2021/5520710	non-relevant (no GRS)

The Basic Characteristics of the Included Studies

All studies were case control in design. The ethnicity of subjects was European in three studies [6,9,10] and Chinese in the other two studies [11,12]. External validation of the GRS was performed in one study only [11]. The type of psoriasis was stated as plaque and guttate in the study by Chen et al. [6] and plaque psoriasis only in the studies by Yin et al. [12], Stawczyk-Macieja et al. [10], and Kisiel et al. [9]. In their study, Yin et al. [11] did not mention the type of psoriasis. In all the studies, the diagnosis of psoriasis was confirmed after examination by dermatologists, though the level of experience was only mentioned by one study [9]. The mean age of onset was markedly higher in the studies by Chen et al. [6] and Kisiel et al. [9] compared to the study by Yin et al. [11] (24.6±15.5 and 26.27±16.32 vs. 21.31±8.69, respectively). The studies by Yin et al. [12] and Stawczyk-Macieja et al. [10] did not state the age at onset. Family history of psoriasis was recorded in three studies [6,9,11] and its frequency ranged from 31.34 to 31.34%. the frequency of female cases was markedly lower in the study by Yin et al. [12] compared to the other studies (5.96 vs. approximately 45%) (Table 2).

The selection of single nucleotide polymorphisms (SNPs) depended on the findings from previous genome-wide association studies (GWAS) [6,9-12], large cohort studies [6,10] as well as meta-analysis and large-scale case-control studies [9]. Calculation of the GRS was performed either using the simple risk alleles count method (count GRS, cGRS) [6,10] or the weighted method (weighted GRS, wGRS) [6,9-12] (Table 3).

**Table 2:** Characteristics of the included studies. N: number; NR: not recorded; SD: standard deviation

Study	Study Design	Study Population	Cohort	N Cases: N Controls	Psoriasis Type	Age Of Onset Mean (SD) years	Female Cases (%)	Family History (%)
Chen et al. 2011 [6]	Case-control	European	Derivation	731: 2,084 + 858 family samples	Plaque & guttate psoriasis	24.6 (15.5)	50.40	31.34
Yin et al., 2014 [12]	Case-control	Chinese	Derivation	7,892: 7,037	Plaque psoriasis	NR	5.96	NR
Yin et al., 2015 [11]	Case-control	Chinese	Derivation	3,621: 3,350	NR	21.31 (8.69)	44.02	31.34
			Validation	712: 723	NR	NR	NR	NR
Stawczyk-Macieja et al., 2016 [10]	Case-control	European	Derivation	148: 146	Plaque psoriasis	NR	46.62	NR
Kisiel et al., 2017 [9]	Case-control	European	Derivation	480: 490	Plaque psoriasis	26.27 (16.32)	46.04	43.33

**Table 3:** Characteristics of the predictive models for psoriasis in the included studies. GRS: genetic risk score; cGRS: count genetic risk score; wGRS: weighted genetic risk score; GWAS: Genome-wide association studies; SNP: single nucleotide polymorphism; HLA: human leucocyte antigen

Study	Used genetic variants	Reference for SNPs	GRS computation	Non-genetic factors included
Chen et al. 2011 [6]	10 SNPs	GWAS and large cohort	Two models: cGRS & wGRS	Intra-European ancestry & gender
Yin et al., 2014 [12]	5 SNPs	GWAS and large cohort	One model: wGRS	Gender
Yin et al., 2015 [11]	14 SNPs	GWAS	Three wGRS models (SNP, HLA and SNP-HLA)	For SNP-HLA GRS model: age, gender, & alcohol consumption
Stawczyk-Macieja et al., 2016 [10]	5 SNPs	GWAS or large cohort	Two models: cGRS & wGRS	None
Kisiel et al., 2017 [9]	38 SNPs	GWAS, meta-analysis, and large-scale case control study	Seven wGRS models: 1-GRS-ALL (all 38 SNPs), 2-GRS-0.1 (19 SNPs with a p < 0.1 in the current study), 3-GRS-N (16 SNPs with uncorrected p-value < 0.05), 4-GRS-B (6 SNPs significantly associated with psoriasis after Bonferroni correction), 5-GRS-HLA (only rs4406273- a proxy for HLA-Cw0602), 6-GRS-N (+) HLA (-) (GRS-N without rs4406273), 7-GRS-N (Subst.) (GRS-N with rs4406273 substituted by rs10484554).	None

Summary of the Included Studies

Chen et al. [6] genotyped 10 psoriasis-risk SNPs in 2815 unrelated case-control samples (731 cases and 2,084 healthy controls) and 858 family samples (633 cases and 225 unaffected subjects) of European ancestry. They selected the risk alleles from GWAS demonstrating P < 5x10-7 and from large cohort studies with evidence of replication at p < 0.05 in at least one independent study. The wGRS was associated with a higher risk than any individual SNP. The psoriasis odds ratio (OR) for top vs. bottom wGRS quartiles was 10.55 (95% confidence interval [CI]: 7.63-14.57, p = 2.010x10-65). The AUC for wGRS was significantly greater than for cGRS (0.720 versus 0.665, p = 2.13x10-8). The AUC for human leucocyte antigen-C (HLA-C) alone (rs10484554) was nearly equivalent to the AUC for all the other nine SNPs combined (66.2% versus 63.8%, p = 0.18). The wGRS was significantly associated with younger age of onset (p = 4.91x10-6) and family history (p = 0.020).

Yin et al. [12] genotyped five SNPs in genes related to IL23/Th17 pathway in 14,929 Han Chinese samples (7,892 plaque psoriasis and 7,037 healthy controls). The wGRS substantially contributed to the risk of psoriasis (p = 3.91× 10−07) and the top quartile (vs. bottom wGRS quartile) was significantly associated with a higher likelihood of psoriasis (OR = 1.22, 95% CI: 1.11-1.34, p = 1.85×10−05).

Another study by Yin et al. [11] developed three wGRS models using 14 susceptibility variants in 8,819 Han Chinese samples (3,621 psoriasis and 3,350 healthy controls). They found that the top GRS quartile versus the lower quartile was significantly associated with a diagnosis of psoriasis (OR = 28.20, P < 2.0×10-16). Significant associations were observed between the GRS, family history, and early age onset of psoriasis. The AUCs for the HLA, SNP, and SNP-HLA models were 0.8343 (95% CI: 0.8255-0.8432), 0.6029 (95% CI: 0.5897-0.6161), and 0.8583 (95% CI: 0.8491-0.8675), respectively. Pairwise comparisons of AUCs revealed a significant difference between HLA and SNP-HLA models (P < 2.20×10-16). The sensitivity and specificity of the SNP-HLA model were 84.70% and 81.70%, respectively. The HLA model showed the highest hazard ratio (HR) of developing early-onset psoriasis (HR: 1.09, 95% CI: 1.07-1.12, p = 1.10×10-12). External validation was conducted on a sample of 1435 (712 psoriasis cases and 723 unaffected individuals), yielding slightly lower AUCs for the three models compared to those obtained from the derivation dataset.

Stawczyk-Macieja et al. [10] assessed five susceptibility loci involved in the immunological response (HLA-C, ERAP1, ZAP70) and the skin barrier function (LCE3, CSTA) between patients with chronic plaque psoriasis (n = 148) and healthy control subjects (n = 146). Two models were created (cGRS and wGRS). The wGRS was more efficient in predicting psoriasis risk than any individual SNPs. The AUC for wGRS was significantly larger than that of the cGRS (0.789 vs. 0.685, p = 0.0002).

Kisiel et al. [9] genotyped 38 SNPs in 480 psoriatic patients and 490 controls. Seven wGRS were created: 1) GRS-ALL (all 38 SNPs); 2) GRS-0.1 (19 SNPs with a p < 0.1 in their study); 3) GRS-N (16 SNPs with uncorrected p < 0.05); 4) GRS-B (six SNPs significantly associated with psoriasis after Bonferroni correction); 5) GRS-HLA (only rs4406273 as a proxy for HLA-Cw0602); 6) GRS-N(+)HLA(-) (GRS-N without rs4406273); and 7) GRS-N(Subst.) (GRS-N with rs4406273 substituted by rs10484554). The analysis of receiver operating characteristics (ROC) curves showed that the GRS-N had significantly higher AUC than those of the GRS-B and HLA-C models (AUC: 0.776 vs. 0.750 and 0.694, P = 1x10−4 and p < 1x10−5, respectively). Adding more SNPs to the model did not improve its discriminatory ability (AUC for GRS-ALL: 0.782, p > 0.05). The OR of diagnosing psoriasis for top vs. bottom GRS-N quartiles was 12.29 (P < 1x10−6). The GRS-N showed a sensitivity of 69.9%, a specificity of 74.1%, and an overall accuracy of 72.0%. The GRS-N was significantly associated with the age of onset (OR for < 40 vs ≥ 40: 0.56, p = 0.014) and family history of psoriasis (OR: 1.83, p = 0.006). Internal validation by dividing the sample into training and set tests showed comparable discriminatory ability.

Results of the Discriminative Power of the Studied Models

Four studies performed logistic regression analysis to assess the contribution of the top versus bottom categories of the GRS to the likelihood of having psoriasis [6,9,11,12]. The association was significant, and the probability of psoriasis increased with the top category of the score in the four studies. The highest OR was reported by Yin et al. [11] (OR: 28.20, 95% CI: 22.95-34.95, P < 2.00×10-16), while the lowest OR was reported by Yin et al. [12] (OR: 1.22, 95 % CI: 1.11-1.34, P = 1.85×10−05) (Table 4).

**Table 4:** Characteristics of the predictive models for psoriasis in the included studies. AUC: area under the receiver operating characteristics curve; CI: confidence interval; GRS: genetic risk score; cGRS: count genetic risk score; wGRS: weighted genetic risk score; HR: hazard ratio; OR: odds ratio; SNP: single nucleotide polymorphism; NR: not recorded; HLA: human leucocyte antigen

Study	Association between GRS and psoriasis (OR)	AUC (95% CI) of model	Association with the age of onset
Chen et al. 2011 [6]	High-low wGRS quartiles OR: 10.55 (95% CI: 7.63–14.57), P = 2.010x10^-65^	cGRS: 0.665 (95% CI: 0.642–0.688), wGRS: 0.720 (95% CI: 0.699–0.741), pairwise comparison P = 2.13x10^-8^	≤ 30 yrs vs > 30 OR: 0.559 (95% CI: 0.433–0.714), P = 4.91x10^-06^
Yin et al., 2014 [12]	OR: 1.22 (95% CI: 1.11–1.34, P = 1.85×10^−05^	NR	NR
Yin et al., 2015 [11] (derivation)	SNP-HLA model: OR = 28.20 (95% CI: 22.95–34.95), P < 2.00×10^–16^	SNP model: 0.6029 (95% CI: 0.5897–0.6161), SNP-HLA model: 0.8583 (95% CI: 0.8491–0.8675), HLA model: 0.8343 (95% CI: 0.8255–0.8432), pairwise comparison HLA vs. SNP-HLA models P < 2.20×10^–16^	SNP model: HR: 1.02 (95% CI: 0.95-1.09), P = 5.50×10-^1^, HLA model: HR: 1.09 (95% CI: 1.07-1.12), P = 1.10×10^-12^, SNP-HLA: HR: 1.08 (95% CI: 1.06-1.11), P = 5.65×10^-12^
Yin et al., 2015 [11] (External validation)	NR	SNP model: 0.5996 (95% CI: 0.5689–0.6303), SNP- SNP-HLA model: 0.8225 (95% CI: 0.7991–0.8458), HLA model: 0.7938 (95% CI: 0.7713–0.8163)	
Stawczyk-Macieja et al., 2016 [10]	NR	cGRS: 0.685, wGRS: 0.789, pairwise comparison p = 0.0002	NR
Kisiel et al., 2017 [9]	GRS-N model: OR for top vs. bottom quartiles 12.29 (P < 1x10^−6^)	GRS-ALL model: 0.782 (95% CI: 0.753-0.811), GRS-0.1: 0.779 (95% CI: 0.750-0.808), GRS-N: 0.779 (95% CI: 0.774-0.805), GRS-B: 0.750 (95% CI: 0.720-0.781), GRS-HLA: 0. 694 (95% CI: 0. 661-0.728), GRS-N (+) HLA (-): 0. 698 (95% CI: 0. 66-0.731), Pairwise comparison: GRS-N vs. GRS-B (0.776 vs. 0.750, P = 1x10^−4^), GRS-HLA (0.776 vs. 0.694, P < 1x10^−5^), GRS-N (+) HLA (-) & GRS-HLA vs. GRS-N (0.694 & 0.698 vs. 0.779, P < 1x10^−5^)	GRS-N: < 40 vs ≥ 40 OR 0.56, p = 0.014

Results of the Discriminative Power of the Studied Models

A total of 15 GRS models were reported by the five included studies. Enough data was reported in the studies to plot 11 models only. Yin et al. [12] did not perform a ROC curve analysis for the GRS, while Stawczyk-Macieja et al. [10] reported only the AUCs without their 95% CI or standard errors. No 95% CI or standard errors were reported for the GRS-N(Subst.) by Kisiel et al. [9]

**Figure 2:**
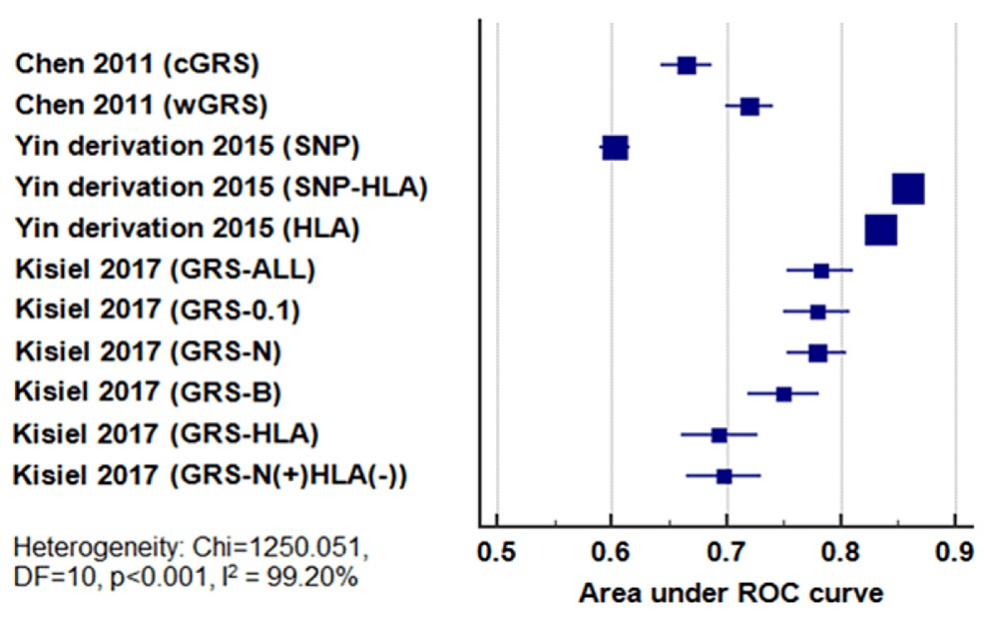
Forest plot for the area under the ROC curve for each genetic risk score as reported by the included studies. ROC: receiver operating characteristics; GRS: genetic risk score; cGRS: count genetic risk score; wGRS: weighted genetic risk score; SNP: single nucleotide polymorphism; HLA: human leucocyte antigen Chen et al., 2011 [6]; Kisiel et al., 2017 [9]; Yin et al., 2015 [11]

The reported AUCs of the 11 GRS models ranged from 0.6029 to 0.8583, with a median value of 0.75. The highest AUCs were those of the SNP-HLA and the HLA GRS models from the study by Yin et al. [11] (Table 4, Figure 2). The reported AUCs varied widely across the various models, presumably depending on the selected SNPs included in each model. Marked heterogeneity was detected by the Cochran Q test (chi-square: 1250.051, p < 0.001) and the I2 index (99.2%). So, pooling of the results of the included models was not performed.

Results of the Association of GRS With Age at Onset

The developed GRS models showed a significant association with the age of onset [6,9,11], except for the SNP model in the study by Yin et al. [11]. Higher GRS was significantly associated with early-onset psoriasis (Table 4).

The Methodological Quality of Included Studies

The results of the assessment of the ROB and applicability using the PROBAST tool can be seen in Table 5. The predictors' section showed uncertain ROB as none of the studies stated whether predictor assessments were made without knowledge of the diagnosis of psoriasis. The ROB in the analysis was high in all the studies because no accounting for the model overfitting and optimism in model performance was provided. Moreover, none of the studies provided sufficient data to judge whether the assigned weights for predictors correspond to the results from the reported multivariable analysis. Also, no calibration of the models was provided in three studies [6,10,12]. External validation was performed in one study only [11]. There was no information regarding the handling of participants with missing data in two studies [10,11]. Consequently, the overall ROB was high for all studies. No concerns of applicability were detected.

**Table 5:** Assessment of the risk of bias of the included studies using the PROBAST tool. PROBAST: Prediction model Risk Of Bias Assessment Tool; ROB: risk of bias; + indicates low ROB/low concern regarding applicability; − indicates high ROB/high concern regarding applicability; and ? indicates unclear ROB/unclear concern regarding applicability

Study	Model	ROB				Applicability			Overall	
		Participants	Predictors	Outcome	Analysis	Participants	Predictors	Outcome	ROB	applicability
Chen et al. 2011 [6]	Derivation	+	?	+	-	+	+	+	-	+
Yin et al., 2014 [12]	Derivation	+	?	+	-	+	+	+	-	+
Yin et al., 2015 [11]	Derivation	+	?	+	-	+	+	+	-	+
	validation	+	?	+	-	+	+	+	-	+
Stawczyk-Macieja et al., 2016 [10]	Derivation	+	?	+	-	+	+	+	-	+
Kisiel et al., 2017 [9]	Derivation	+	?	+	-	+	+	+	-	+

Discussion

Summary of Main Results

The results of this systematic review show that GRS for psoriasis can accurately discriminate between psoriasis and normal skin. These results indicate the potential of using GRS for the prediction of psoriasis. Fifteen models from five studies were included in the present systematic review. Two GRS models were calculated using the count method [6,10], while the remaining models were weighted GRS [6,9-12]. The discriminatory power of the wGRS models was superior to the cGRS models [6,10]. This could be attributed to the wGRS accommodating differences in SNPs influence on genetic predisposition to the disease [6,21].

Previous research showed that the HLA-Cw*0602 allele is significantly associated with younger age at onset, a severer disease course, and the guttate phenotype [22,23]. Twelve out of the fifteen models included SNPs related to the HLA-Cw*0602 allele. The models lacking the HLA-Cw*0602 allele included the model by Yin et al. [12], the SNP model by Yin et al. [11], and the GRS-N(+)HLA(-) model by Kisiel et al. [9]. The addition of other SNPs to the HLA-C models increased their discriminatory power to a strong degree in the study by Kisiel et al. [9], but to a slight degree in the studies by Yin et al. [11] and Stawczyk-Macieja et al. [10]. Chen et al. found that the AUC for HLA-C was slightly higher than that for the GRS without HLA-C (0.662 vs. 0.638) and significantly lower than AUC for the GRS with HLA-C (0.720). In three studies [6,10,11], the discriminatory ability of the GRS was determined mainly by HLA-C, as observed by the significantly higher AUC for HLA-C than AUCs for the non-HLA-C SNPs. However, Kisiel et al. [9] reported a comparable discriminative power for the GRS-N (+) HLA (-) and GRS-HLA models. This could be explained by the differences in SNPs selected in the studies and their contribution to the discriminatory power of the GRS model. The lower OR for the model developed by Yin et al. [12] may be attributed to the non-inclusion of HLA-C SNPs in their model.

The association of higher GRS with younger age at onset could also be explained by the inclusion of SNPs that have been described with early-onset psoriasis in GWAS, notably the HLA-C locus [24]. This may suggest a stronger genetic basis in early-onset versus late-onset psoriasis. Another probable explanation is that early-onset psoriasis is far more prevalent than late-onset psoriasis; thus, this may lead to the under-representation of late-onset psoriatic patients in GWAS studies. This point could be clarified by conducting a GWAS with a special focus on late-onset psoriatic patients. Such a GWAS may reveal new loci that are associated with late-onset disease and help improve the prediction of those patients. The calculation of commonly used diagnostic accuracy measures (such as sensitivity, specificity, and predictive values) was not feasible as there was no sufficient reported data for most models.

Overall Completeness, Applicability, and Quality of the Evidence

All the included studies carried a high ROB, particularly regarding the analysis domain. External validation was conducted by Yin et al. [11] only. Internal validation was inappropriately conducted by Kisiel et al. [9] by dividing the sample into larger and smaller sets. Appropriate internal validation methods include bootstrapping and cross-validation. In addition, none of the studies mentioned masking the GRS assessors regarding the diagnosis of patients or the examined samples. This warrants the conduction of future studies that avoid the ROB demonstrated by the currently available studies.

Agreements and Disagreements With Other Studies or Reviews

A previous systematic review by Burden-Teh et al. [3] critically appraised the studies that had the primary research aim to develop or validate diagnostic criteria for psoriasis. The systematic review was not confined to the use of GRS as it included other diagnostic tests histological examination and imaging. The review by Burden-Teh et al. [3] included only two studies out of the five analyzed in the present review and did not discuss the discriminatory power of the various models. In addition, they included studies that were excluded from the current review due to a lack of clarity in model calculation and the ethnicities of participants.

## Conclusions

The results of this systematic review show that GRS are promising tools for the prediction of psoriasis, with fair to good accuracy. However, the high ROB of the included studies and the lack of validation or suggested methods of application in routine clinical practice necessitate the launching of further research to identify the most accurate combination of loci and validate the scores in variable ethnicities. In addition, future GWAS should focus on late-onset psoriatic patients to identify the loci that are associated with late-onset disease.

Prediction of psoriasis continues to be a critical priority for medical professionals and biomedical researchers as well as patients. The expanding sources of genetic data and biological markers should be coupled with clinical information and the established scoring systems to develop innovative methods to enhance diagnosis of psoriasis.
